# TRI Microspheres prevent key signs of dry eye disease in a murine, inflammatory model

**DOI:** 10.1038/s41598-017-17869-y

**Published:** 2017-12-13

**Authors:** Michelle L. Ratay, Stephen C. Balmert, Abhinav P. Acharya, Ashlee C. Greene, Thiagarajan Meyyappan, Steven R. Little

**Affiliations:** 1 Department of Bioengineering, University of Pittsburgh, Pittsburgh, PA 15261 USA; 2 Department of Chemical Engineering, University of Pittsburgh, Pittsburgh, PA 15216 USA; 3 Department of Immunology, University of Pittsburgh, Pittsburgh, PA 15213 USA; 4 Department of Ophthalmology, University of Pittsburgh, Pittsburgh, PA 15213 USA; 5 Department of Pharmaceutical Science, University of Pittsburgh, Pittsburgh, PA 15261 USA

## Abstract

Dry eye disease (DED) is a highly prevalent, ocular disorder characterized by an abnormal tear film and ocular surface. Recent experimental data has suggested that the underlying pathology of DED involves inflammation of the lacrimal functional unit (LFU), comprising the cornea, conjunctiva, lacrimal gland and interconnecting innervation. This inflammation of the LFU ultimately results in tissue deterioration and the symptoms of DED. Moreover, an increase of pathogenic lymphocyte infiltration and the secretion of pro-inflammatory cytokines are involved in the propagation of DED-associated inflammation. Studies have demonstrated that the adoptive transfer of regulatory T cells (Tregs) can mediate the inflammation caused by pathogenic lymphocytes. Thus, as an approach to treating the inflammation associated with DED, we hypothesized that it was possible to enrich the body’s own endogenous Tregs by locally delivering a specific combination of Treg inducing factors through degradable polymer microspheres (**TRI** microspheres; **T**GF-β1, **R**apamycin (Rapa), and **I**L-2). This local controlled release system is capable of shifting the balance of Treg/T effectors and, in turn, preventing key signs of dry eye disease such as aqueous tear secretion, conjunctival goblet cells, epithelial corneal integrity, and reduce the pro-inflammatory cytokine milieu in the tissue.

## Introduction

Dry eye disease (DED) is a common ocular disorder affecting millions of individuals with an average annual direct medical cost of $3.84 billion in the United States^1–4^. A myriad of risk factors have been associated with DED including: autoimmune diseases (rheumatoid arthritis, Sjögren’s Syndrome), thyroid disease, hormonal changes, and aging^4^. Typically, these risk factors can lead to symptoms such as ocular irritation, reduced visual acuity, and tear hyperosmolarity^5,6^. In turn, the increase in tear hyperosmolarity leads to ocular surface inflammation and tissue destruction^7^.

Recent studies strongly suggest that the ocular surface inflammation is the result of pathogenic effector T cells secreting pro-inflammatory cytokines^8–14^. Accordingly, treatments have been developed that intend to suppress the inflammatory response through the administration of anti-inflammatory treatments such as corticosteroids^15^. However, long-term topical use of corticosteroids have been implicated in conditions such as glaucoma and retinopathy^16^. An ideal treatment for DED would address the underlying inflammation mediated by the pathogenic effector T cells without serious, negative side effects.

One method that the body uses to decrease local function/prevalence of effector T cells is through enhancing the prevalence and/or function of immunosuppressive cells (regulatory T cells) Tregs to resolve inflammation^12,17^. Specifically, prevalent, naïve CD4^+^ T cell populations in the periphery are capable of differentiating into functional Tregs under the direction of a subset of antigen presenting cells known as tolerogenic dendritic cells (tDCs)^18^. tDCs induce differentiation of Tregs (in part) through the secretion of a combination of IL-2 and TGF-β cytokines^19,20^. However, the maintenance of Tregs is somewhat more complex and depends on a local microenvironment that is not only favorable to differentiation of Tregs, but also unfavorable to differentiation into other effector T cells^21^. One method of creating such a local microenvironment is through administration of the small molecule, rapamycin. Rapamycin (Rapa) is an mTOR inhibitor that can suppress the generation and proliferation of effector T cells^22^. We have previously demonstrated that sustaining the presence of TGF-β, Rapamycin and IL-2 using degradable microspheres was able to induce/differentiate naive CD4^+^ T cells into FoxP3^+^ Tregs *in vitro* with high efficiency^22^.

Here we describe the *in vivo* application of T-Reg Inducing (TRI) microspheres (MS) in a model of murine dry eye disease. Data suggests that this drug-delivery strategy can influence local Treg numbers and, in turn, prevent key signs of DED. Application of this new strategy could provide a potential avenue for new types of immune based treatments for DED that influence the body’s own cells to address destructive inflammation^23,24^.

## Methods

### Fabrication of Microspheres

TGF-β and IL-2 microspheres were fabricated using a double emulsion- evaporation technique. For the TGF-β microspheres, Poly (lactic-co-glycolic) acid (PLGA-50:50 lactide:glycolide, acid terminated) (MW:7,000-17,000) (viscosity: 0.16–0.24 dL/g, 0.1% (w/v) in chloroform) (Sigma Aldrich, MO) and PEG-PLGA (PolySciTech, IN) was used to encapsulate rh-TGF-β (PeproTech, NJ). Specifically, 170 mg of PLGA and 30 mg of PEG-PLGA was dissolved in 4 ml of DCM (Sigma Aldrich, MO). Then 200 µl of aqueous solution containing 10 µg of rh-TGF-β was added to the polymer DCM mixture. The mixture was sonicated using a sonicator (Vibra-Cell, Newton, CT) for 10 sec. at 25% amplitude. Next, this emulsion was then mixed with 60 ml of 2% polyvinyl-alcohol (PVA, MW ~25,000, 98% hydrolyzed; PolySciences) and homogenized (L4RT-A, Silverson, procured through Fisher Scientific) at 3,000 rpm for 1 min. The homogenized mixtures were then added to 80 ml of 1% PVA on stir plate and left for 3 hours in order for the DCM to evaporate. After 3 hours, the microparticles were centrifuged (200 g, 5 min, 4 °C), washed 5 times with deionized water, and lyophilized for 48 hours (Virtis Benchtop K freeze dryer, Gardiner, NY).

For the IL-2 microspheres, 200 mg of PLGA (PLGA-50:50 lactide:glycolide, acid terminated) (MW:7,000-17,000) (viscosity: 0.16–0.24 dL/g, 0.1% (w/v) in chloroform) (Sigma, Aldrich, MI) was dissolved in 4 ml of DCM. Subsequently, 5 µg of IL-2 and 150 µl (R&D Systems, Minneapolis MN) of deionized water was added to the organic phase. Next, the two phases were emulsified using a sonicator probe (Vibra-Cell, Newton, CT) at 25% amplitude for a period of 25 seconds. Then this emulsion was mixed with 60 ml of 2% polyvinyl-alcohol (PVA, MW ~25,000, 98% hydrolyzed; Polysciences) with 51.66 millimoles of NaCl and homogenized (L4RT-A, Silverson, procured through Fisher Scientific) at 3,000 rpm for 1 min. This secondary emulsion was then then added to 80 ml of 1% PVA on stir plate and stirred for 3 hours. After finishing stirring, the microparticles were centrifuged (200 g, 5 min, 4 °C), washed 5 times with deionized water, and lyophilized for 48 hours (Virtis Benchtop K freeze dryer, Gardiner, NY).

The rapamycin (rapa) microspheres were fabricated using a single emulsion-evaporation technique due to the hydrophobic nature. Rapamycin (Sigma Aldrich, MO) was dissolved in DMSO (Sigma, Aldrich, MO) at 10 mg/ml. Then 200 mg of PLGA (Sigma Aldrich, MI) was dissolved in 4 ml of DCM. Next, 100 µl of rapamycin (10 mg/ml) was added to the polymer/DCM mixture. The solution was then homogenized with 60 ml of 2% PVA at 3,000 rpm for 1 min. After homogenizing, the emulsion was then added to 80 ml of 1% PVA and stirred for 3 hours. At the end of stirring, microspheres were washed 5 times with deionized water and lyophilized for 48 hours.

### Characterization of Microspheres

The morphology of the microspheres were characterized using scanning electron microscopy (JEOL, JSM-6330F, Peabody, MA) and volume impedance measurements were performed on a Beckman Coulter Counter (Multisizer-3, Beckman Coulter, Fullerton, CA). The release assay of the IL-2, TGF-β, and rapamycin was completed by incubating 10 mg of microspheres in 1 ml of phosphate buffered saline (PBS) and 1% BSA, which was placed onto a rotator at 37 °C. The supernant was sampled at different time intervals and the TGF-β and IL-2 release profiles were quantified using an enzyme-linked immunosorbent assay (ELISA) (R&D Systems, Minneapolis, MN). The release profile of rapamycin microspheres was determined using UV-vis spectroscopy, and the release media contained 0.2% Tween-80 in PBS (absorbance at 278 nm).

### Mice

Female Balb/c mice aged 6–8 weeks were used in this experimental study. (Charles Rivers Laboratories, Wilmington, MA). The Institutional Animal Care and Use Committee, University of Pittsburgh approved all murine experiments. All methods were performed in accordance with the relevant guidelines and regulations.

### Murine DED model and treatment

Dry eye disease was induced using 10 mg/ml of Concanavalin A (ConA) (Sigma Aldrich, St. Louis, MO) in phosphate buffered saline solution (PBS) was injected into the lacrimal glands with a 28.5 gauge needle using a dissecting microscope^25,26^. The controls for examining the effects of the TRI MS included Blank (unloaded) or TRI MS (25 mg/ml). The TRI microspheres are a combination of all three microspheres (rapamycin microspheres, IL-2 microspheres, and TGF-β microspheres) individually combined together and injected with ConA. Approximately, 8.33 mg of Rapamycin microspheres, 8.33 mg of IL-2 microspheres, and 8.33 mg of TGF-β microspheres (total 25 mg/ml), which were combined with a PBS solution of ConA (10 mg/ml) (total volume injected into lacrimal gland is 20 µl). (Olympus SZX10, Waltham, MA).

### Suppression of Tregs via the administration of anti-GITR

In order to identify the role of Tregs with the administration of our preventative treatment, the function of Tregs were inhibited using anti-GITR (DTA-1) (BioXCell, Lebanon, NH) via an intraparietal injection of (500 µg per mouse) 1 day after injecting the ConA and TRI MS^27^.

### Tear Production

Phenol red cotton threads were utilized to measure tear production. (Oasis Medical, San Dimas, CA). The thread was placed in the lateral canthus of the eye for a period of 60 seconds, and the amount of wetting on the thread was measured using a dissecting microscope (Olympus SZX10, Waltham, MA)^25^.

### Corneal Fluorescein Staining

Fluorescein stain (1% solution) was applied to the conjunctival sac. The surface of the cornea was examined using a dissecting microscope (Olympus SZX10, Waltham, MA). The scoring of staining was completed by a masked ophthalmologist, and scored 0 for no staining, score 1 for a quarter of staining, score of 2 for less than a half, score of 3 for half, and 4 for more than half of the eye.

### Ocular Histology

At the conclusion of the study, the eyes were exenterated and fixed in 10% neutral buffered formalin. Sections were prepared at approximately 5 µm and stained with Periodic Acid Schiff (PAS) in order to examine goblet cell density. Histological sections were scanned and quantified using a Zeiss Axio Scan. Z1 (Thornwood, NY) and Pannoramic Viewer software (3D HISTECH Ltd.).

### qRT-PCR

Total RNA was extracted from multiple excised lacrimal glands using TRI-reagent (Molecular Research Center, Cincinnati, OH), and quantified using a NanoDrop 2000 (Thermo Scientific). For the reverse transcriptase assay, 2 μg RNA was converted to cDNA using a QuantiTect Reverse Transcription Kit (Qiagen, Valencia, CA). Quantitative real-time PCR was then performed using VeriQuest Probe qPCR Mastermix (Affymetrix, Santa Clara, CA), (Thermo Scientific) specific for (IFN-γ:Mm01168134_m1, FAM-MGB dye), (IL-2:Mm00434256_m1, FAM-MGB dye), (IL-6:Mm00446190_ml, FAM-MGB dye), and (Gusb: Mm01197698_m1, VIC-MGB PL dye, endogenous control). Duplex reactions (target gene + GUSB) were run and analyzed on a StepOnePlus Real-Time PCR System (Applied Biosystems, Carlsbad, CA). Relative fold changes of IFN-γ, IL-6 and IL-2 expression were calculated and normalized based upon the 2^−ΔΔCt^ method, with the Saline group as the untreated control.

### Immunofluorescence of the lacrimal gland

At the end of the study, lacrimal glands were excised from the mice. Lacrimal glands were fixed with 4% PFA overnight, followed by cryoprotection through incubation in 30% sucrose overnight, and lastly embedded in O.C.T. medium. The cyrosections were obtained at 7 μm thick and stained with fluorescent antibodies. Specifically, 7 μm sections were blocked with 5% normal donkey serum and 1% Tween20 in PBS. Blocked sections were incubated overnight at 4 °C with rat anti-FoxP3 (FJK-16s; eBio) and rabbit anti-CD3 (SP7, monoclonal rabbit IgG; Abcam, Cambridge, MA). The sections were then incubated with a secondary antibody, Alexa Fluor 594 donkey anti-rat IgG (ThermoFisher Scientific Waltham, MA) and Alexa Fluor 647 donkey anti-rabbit IgG (Jackson ImmunoResearch Laboratories, West Grove, PA) for 1 hour at room temperature and then mounted using Fluoroshield mounting medium with DAPI (Abcam, Cambridge, MA). The images were captured using a Zeiss Axio Scanner Z.1. Only positively stained cells overlapping DAPI (nuclei) were quantified.

### Statistical Analysis

Data expressed as mean ± S.D. Comparisons between multiple treatment groups were performed using one-way ANOVA, followed by Bonferroni multiple comparisons, and p ≤ 0.05 was considered statistically significant. The PCR data expressed as mean ± SEM was analyzed utilizing a t-test with Welch correction, and p ≤ 0.05 was considered statistically significant. Statistical tests were performed using GraphPad Prism Software 6.0 (GraphPad Prism, San Diego, CA).

## Results

### Characterization of TRI MS: IL-2, TGF-β1 and Rapamycin

TGF-β microspheres (MS) were reformulated from what was previously described [refs^28–31^] to eliminate the 20-day initial lag phase of release in the prior formulation. The new formulation of TGF-β MS contains a PEG-PLGA diblock copolymer (4 wt%, Mn ~5 kDa), which accelerated release by increasing matrix swelling, and the ester-terminated PLGA helped to minimize the electrostatic interactions between the PLGA polymer and the positively charged protein^32^. After measuring the release of TGF-β, the surface morphology of the microspheres was characterized using scanning electron microscopy (SEM). The representative SEM images indicate that the rapamycin and IL-2 MS possessed similar surface morphology and release behavior as previously reported (Supplemental Fig. 1)^22^. SEM images reveal spherical non-porous PLGA-based rapamycin microspheres (Supplemental Fig. 1). IL-2 MS exhibit surface porosity and a high initial burst followed by a slow continuous release for the length of the experimental study, as previously described (Supplemental Fig. 1)^22^. The newly fabricated TGF-β microspheres contained an uneven surface morphology, similar to a previous report utilizing PEG and PLGA microcapsules^28^. The average size of TRI MS was 12 µm (rapamycin), 19 µm (IL-2), and 17 µm (TGF-β) as determined by Coulter Counter (volume impedance method).

### TRI MS Prevent Loss of Aqueous Tear Production

**Figure 1:**
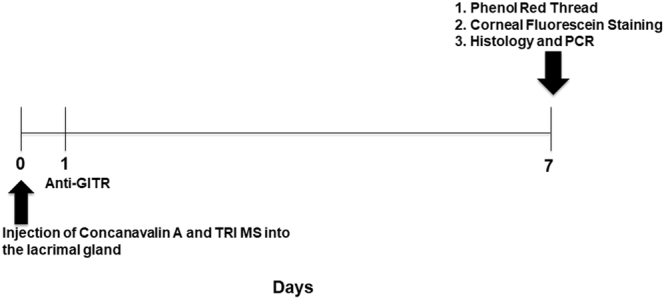
TRI microspheres for the prevention of inflammation associated with Dry eye Disease (DED) in mice. A timeline for the experimental murine model of inflammation induce via Concanavalin A.

**Figure 2:**
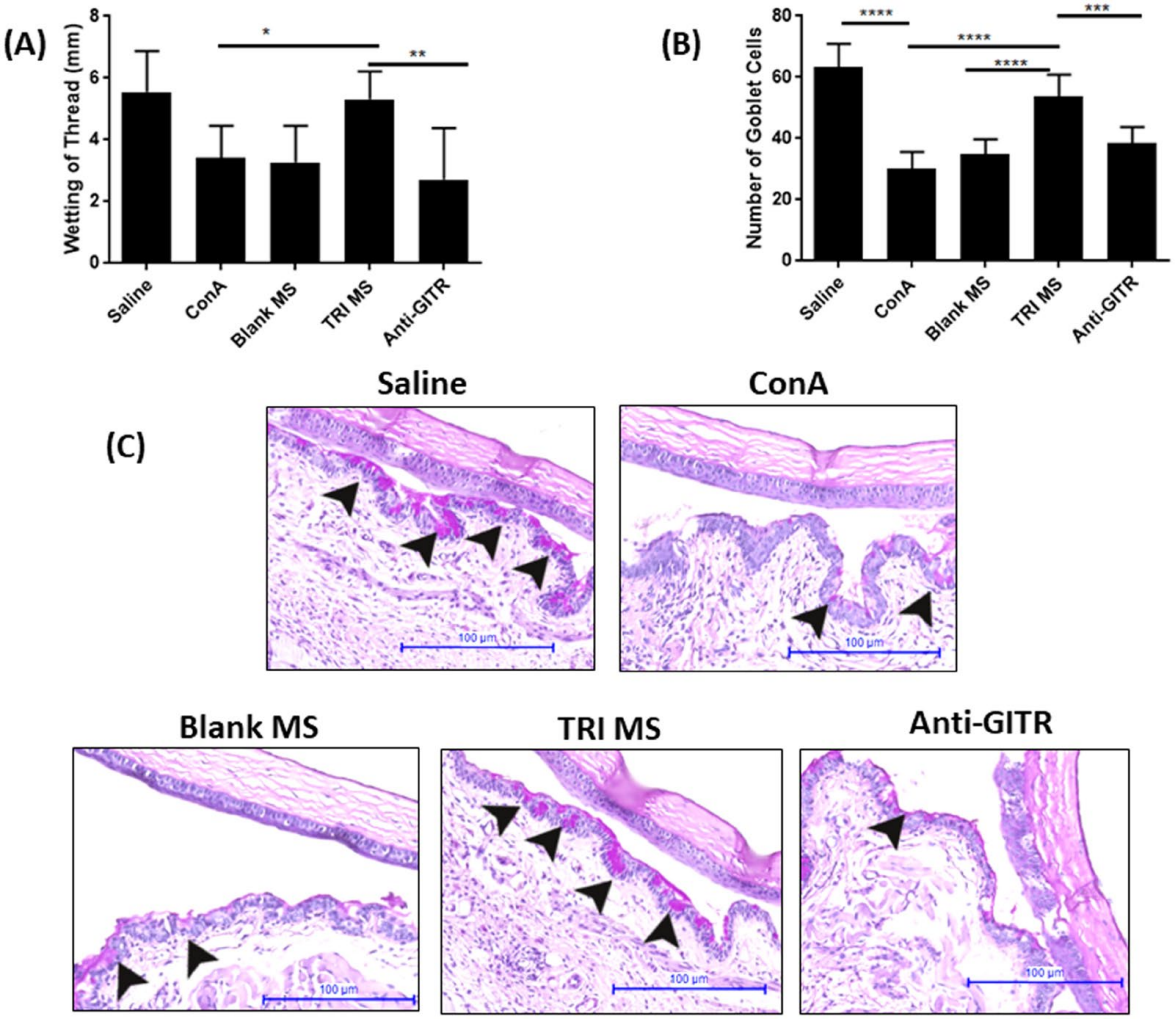
TRI MS prevent clinical signs of inflammation associated with DED. (**A**) Wetting of phenol red threads were measured in millimeters using a dissecting microscope (n = 6) shown as mean ± S.D. (**B**) Representative images of histological sections of the eyes (20X) were quantified to identify differences in the TRI MS group compared to the diseased groups and non-diseased group (100 µm scale bar). (**C**) Goblet cells shown are the pink/purple (Periodic Acid Schiff stained) cells located in the conjunctiva labeled with arrows and the groups are shown as mean ± S.D. *p ≤ 0.05; **p ≤ 0.01; ***p ≤ 0.001, ****p ≤ 0.0001.

To investigate whether TRI MS were capable of preventing key signs of dry eye disease, we first examined aqueous tear secretion^25^. Concanavalin A (ConA) was injected into the lacrimal gland to induce DED, and for TRI MS or Blank MS treatment groups, MS were incorporated in ConA injections (Fig. 1). One week following the administration of ConA with either Blank MS or TRI MS, phenol red thread testing was performed to evaluate tear secretion. The administration of ConA alone (diseased) considerably reduced tear production as compared to an injection of Saline (non-diseased) (Fig. 2A). Notably, tear secretion was restored to non-diseased levels in DED mice treated with TRI MS, while administration of ConA + Blank MS (unloaded) had no noticeable effect on tear production in mice (Fig. 2A). In order to identify whether all three factors (TRI MS) were required to prevent loss of aqueous tear production, mice were treated with individual microsphere formulations alone (Rapa, TGF-β, or IL-2) or combinations of two microsphere formulations (Rapa + TGF-β; Rapa + IL-2; TGF-β + IL-2). Notably, the individual microspheres alone and the combinations of two microsphere formulations were unable to restore tear production inhibited by ConA (Supplemental Fig. 2), suggesting that therapeutic efficacy required the delivery of the TRI MS to prevent the loss of tear production.

### Goblet Cell Density Maintained with the Administration of TRI MS

Mucin is a key component associated with a healthy tear film, which is produced by goblet cells located in the conjunctiva^29,30^. As the density of goblet cells are diminished this can contribute to an unstable tear film and lead to ocular surface destruction^30^. Upon examination of ocular tissue histology, we observed a substantial decrease in the density of Periodic Acid Schiff (PAS)-stained goblet cells (pink/purple cells in conjunctiva epithelium layer) in the ConA group as compared to the Saline group (Fig. 2B,C)^25,26,29,31,33^. Treatment with TRI MS led to maintenance of goblet cell density, unlike mice with ConA-induced DED (Fig. 2B). Interestingly, the individual microspheres alone and the combinations of two microsphere formulations were unable to prevent goblet cell loss (Supplemental Fig. 3). Overall, histological sections revealed that TRI MS treatment markedly inhibited ConA-induced attenuation of goblet cells.

### Corneal Fluorescein Staining Reduced with TRI MS

**Figure 3:**
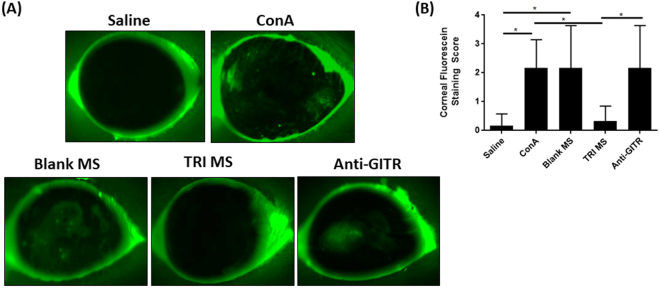
TRI MS reduce ocular surface staining. (**A**) Representative images of corneal fluorescein staining. (**B**) Clinical corneal fluorescein staining scores of the ocular surface on a scale of (0–4) (n = 6) shown as mean ± S.D. *p ≤ 0.05.

To determine the health of the ocular surface, corneal fluorescein staining was performed, with the degree of punctate staining as an indicator of disease severity^34^. Fluorescent images of the ocular surface were captured and scored by a masked ophthalmologist on a scale of 0 to 4, with 0 corresponding to no staining, and 4 corresponding to staining on more than 50% of the cornea, as seen in (Fig. 3A). The ocular staining score was considerably lower for the Saline and TRI MS groups as compared to the ConA + Blank MS group (Fig. 3B). We also examined eyes from mice treated with ConA plus individual microsphere formulations alone or combinations of two microspheres. Neither individual microspheres alone and combinations of two microsphere formulations, were able to reduce corneal fluorescein staining to the same extent as the TRI MS treatment (Supplemental Fig. 2), suggesting that local administration of TRI MS is necessary to restore ocular surface health that is impaired by ConA.

### TRI MS Decrease Pro-Inflammatory Cytokines

**Figure 4:**
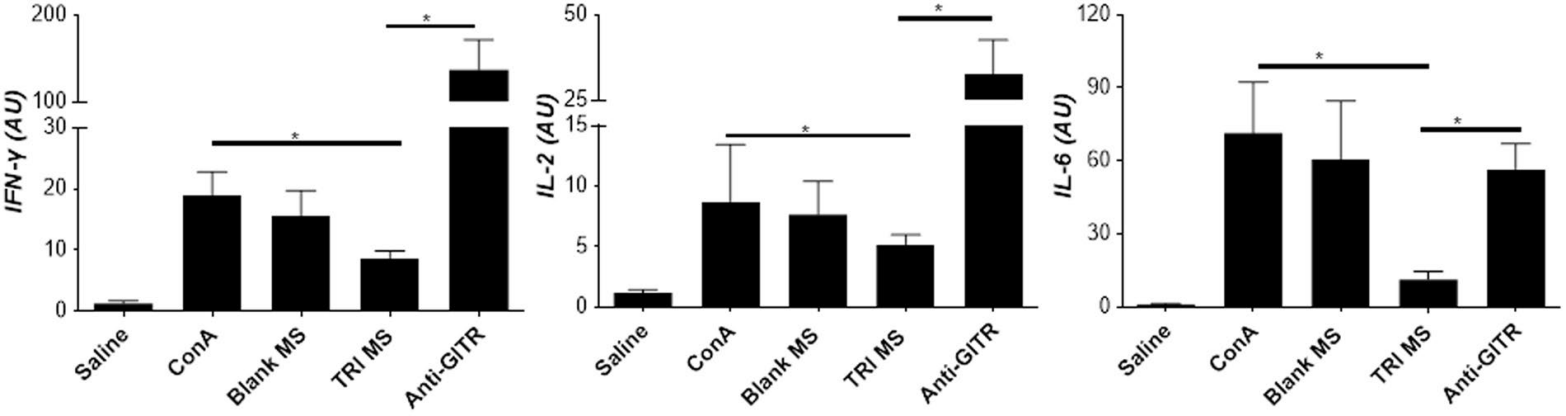
Administration of TRI MS reduces levels of cytokines in the lacrimal gland shown as mean ± SEM. *p ≤ 0.05.

**Figure 5:**
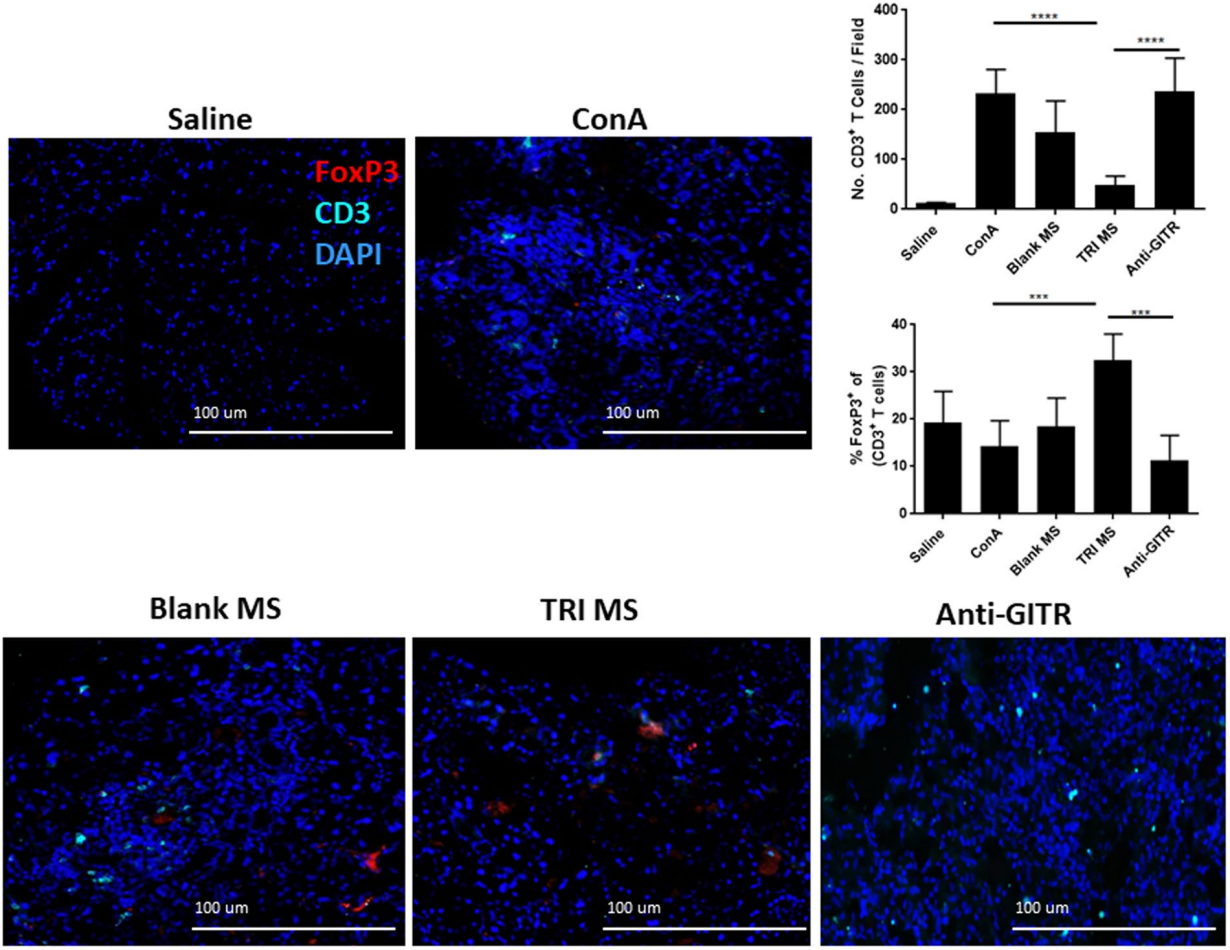
Representative lacrimal gland fixed frozen cryosections (10X magnification) stained for T-cells (CD3^+^ T cells-Cyan), Regulatory T-cells (FoxP3^+^ T cells - Red), and nuclei (DAPI-blue). Scale bars are 100 μm. Quantification of lacrimal gland T cells per imaged field and % Treg, based on IHC images with FoxP3 staining (n = 4–5 per group) as shown as mean ± S.D *p ≤ 0.05; **p ≤ 0.01; ***p ≤ 0.001.

Several cytokines in the local milieu of the lacrimal glands was examined following treatment. Notably, while ConA induced expression of pro-inflammatory IFN-γ, IL-6, and IL-2 in the lacrimal gland (Fig. 4), TRI MS treatment substantially reduced expression of each of these cytokines, compared to the administration of ConA alone (Fig. 4)^25,26^. The relative expression of pro-inflammatory cytokines in the lacrimal glands can be correlated with infiltration of CD3^+^ T cells, which was increased in the ConA + Blank MS group and reduced with TRI MS treatment (Fig. 5)^35^. Together these data indicate that the TRI MS treatment was able to reduce the ConA-induced expression of pro-inflammatory cytokines in the lacrimal gland tissue.

### TRI MS Increase the Percentage of FoxP3^+^ Tregs of overall CD3^+^ T cells in the Lacrimal Gland

In order to examine the local immune environment of T-cells in the lacrimal gland, we performed immunofluorescence staining of lacrimal gland sections with anti-CD3 and anti-FoxP3 monoclonal antibodies. While there were fewer total numbers of infiltrating CD3^+^ T cells with TRI MS treatment, there was a larger ratio of those FoxP3^+^ Tregs of overall CD3^+^ T cells, compared to the ConA alone and ConA + Blank MS groups (Fig. 5). Collectively, this data suggest that the sustained delivery of TRI MS is capable of locally enriching Treg populations as compared to effector T cells in the lacrimal gland tissue.

### Suppression of Tregs via Administration of Anti-GITR

An agonistic antibody (DTA-1) specific for GITR (glucocorticoid tumor necrosis factor) was administered 1 day after the injection of the ConA and TRI MS to determine whether the prevention of dry eye symptoms are mediated by the expanded Treg population^36^. Monoclonal antibody anti-GITR (DTA-1) acts to systemically attenuate the suppressive function of Tregs by inhibiting the ability of conventional T cells to be suppressed by Tregs^37–39^. Mice were administered anti-GITR, one day after ConA and TRI MS. The anti-GITR group (with TRI MP administration) exhibited restored pathological features of DED as indicated by the decrease of aqueous tear secretion (Fig. 1A), reduction of goblet cells in the conjunctiva (Fig. 2B,E) and increase of fluorescein staining (Fig. 3C,D) as compared to the TRI MS group without anti-GITR. Moreover, with the administration of anti-GITR, expression of pro-inflammatory cytokines were considerably increased in the lacrimal gland as compared to the TRI MS with no anti-GITR (Fig. 5).

## Discussion

Dry eye disease (DED) is an ocular disorder that accounts for approximately 25 percent of patient visits to an eye care specialist^40^. Due to the prominent number of patients suffering from symptoms of DED, a number of investigations are examining the underlying cause of the disease^4,5,11^. Specifically, DED is thought to be mediated by CD4^+^ T cells^41,42^. Hallmarks of CD4^+^ T cell-mediated inflammation associated with DED include epithelial apoptosis, abnormal tear film composition and an increase of pro-inflammatory cytokines within the ocular tissue^43,44^. Furthermore, these claims have been substantiated adoptive transfer of pathogenic CD4^+^ T cells from a mouse with DED-like symptoms to a normal recipient, leading to the development of DED^10^. Conversely, when a subset population of CD4^+^ T cells known as regulatory T cells (Tregs) were adoptively transferred to a mouse with DED, immune-mediated ocular surface inflammation was attenuated^45^. Moreover, the local recruitment of Tregs to the lacrimal gland utilizing controlled release of CCL22 (chemokine) effectively prevented signs of DED^46^. Notably, however, Tregs can represent low total numbers of T-cells in the periphery, which could possibly make it non-trivial to achieve enough functional Treg to shift the homeostatic balance^47^. For this reason, we hypothesized that inducing (differentiating) Tregs from a more prevalent, naive CD4^+^ population could also be a viable strategy that was worth exploration.

Typically, signs of DED in patients can include ocular dryness due to a reduction in tears, which can lead to a decrease of visual acuity^30,48^. Within the tears there are three main components that support the ocular surface; water, mucin, and lipid^49^. All of these three components play an integral role in lubricating the ocular surface and maintaining health^50^. As ocular dryness ensues, a lack of lubrication and epithelial surface protection can result^51^. Due to the integral role tears play in maintaining a healthy ocular surface, tear secretion was evaluated to determine whether the preventative treatment (TRI MS) decreased tear loss thereby subsequently preserving ocular lubrication. Our data suggest there was a substantial reduction of tears in the ConA alone as compared to the Saline (Fig. 2), as expected^25,26,46^. Notably, the TRI MS considerably prevented the reduction of tears as compared to the ConA alone group (Fig. 2). However, when either the individual microsphere formulations alone (Rapa, TGF-β, IL-2) or the combination of two microsphere formulations (Rapa + TGF-β; Rapa + IL-2; TGF-β + IL-2) were administered, there was no substantial restoration of tear production observed (Supplemental Fig. 2). These data suggest the combination of all three factors (TRI MS) are required to achieve prevention of tear loss.

In addition to tear production, we also investigated the effects of the treatment on goblet cells, which produce the tear film (mucin)^52^; a key component that provides a protective and stabilizing function for the ocular surface^53^. The histological sections of the eye tissue were examined to identify if goblet cells were preserved with the administration of TRI MS. Notably, there was a considerable preservation of goblet cells in the TRI MS group as compared to the ConA alone and ConA + Blank MS groups (Fig. 2). This may be due to reduced expression of IFN-γ in lacrimal glands of mice treated with TRI MS, compared to the ConA alone treated mice (Fig. 4), as increased expression of IFN-γ has been attributed to the sustained proliferation of CD4^+^ effector T cells (Th1 cells)^41,43^. For example, mice with DED exhibit increased frequencies of the pro-inflammatory Th1 cytokine IFN-γ, which can infiltrate the conjunctiva, causing a reduction of goblet cells^18^. Notably, previously published data suggest that the overall number of goblet cells were not affected in IFN-γ knock out mice with DED, demonstrating the potential specific involvement of IFN-γ in DED pathogenesis^54^. Indeed, the protective effects of TRI MS may be a result of reduced infiltrating Th1 cells in general, or a reduction in IFN-γ specifically (Fig. 4)^44^.

As goblet cells undergo apoptosis, potentially due to IFN-γ, the composition of the tear film can become abnormal, which may lead to a disruption of the corneal epithelial tissue^51^. For this reason, an ocular staining test was performed to determine if the reductions in tear secretion and goblet cell density corresponded to an increase of corneal fluorescein staining^55^. Specifically, the increase in corneal staining may be due to the fluorescein dye remaining in areas left by desquamated epithelial cells^55^. As expected, corneas from the ConA alone and ConA + Blank MS groups showed a considerable increase of corneal fluorescein staining compared to the Saline and TRI MS groups (Fig. 3).

Previous reports have shown that a potential explanation for the reduction of fluorescein staining may be reduced levels of pro-inflammatory cytokines^11,56^. To investigate whether the reduction in corneal epithelial destruction in our studies corresponded with a decrease in pro-inflammatory cytokines, qRT-PCR was performed on the lacrimal gland tissue to detect changes in expression of IL-2, IL-6 and IFN-γ. In addition to the better-known inflammatory function of IL-2 and IFN-γ, there has been observed increases of the pro-inflammatory cytokine IL-6 in dry eye patients^11^. As expected, IL-2 and IFN-γ levels were considerably decreased in the TRI MS as compared to the ConA alone and ConA + Blank MS groups, which may be due to TRI MS mitigating the effects of ConA causing inflammation^26,57^. A decrease of IL-6 expression levels was also detected in the TRI MS as compared to ConA alone group. Overall, there was a substantial difference between the ConA alone and ConA + Blank MS and TRI MS groups levels of pro-inflammatory cytokines within the lacrimal gland. These results likely correlate to the observed decrease of CD3^+^ T cells in the TRI MS as compared to the ConA alone group (Fig. 5).

If the shift in the pro-inflammatory milieu of the ocular tissue were a result of the TRI MS expanding the expression of FoxP3^+^, then it would be expected that there would be a greater number of Tregs (or at least an increase in the ratio of Treg-to-effector T-cells) in the lacrimal gland tissue. Accordingly, immunofluorescent staining of total CD3^+^ T cells and FoxP3^+^ Tregs was performed on the lacrimal gland tissue. We indeed observed a considerable increase in total numbers of CD3^+^ T cells in the lacrimal glands of ConA alone and ConA + Blank MS groups, as compared to the Saline and TRI MS groups (Fig. 5). We also observed a considerable increase in the ratio of Treg-to-effector T-cells in the TRI MS as compared to the ConA alone and ConA + Blank MS groups (Fig. 5), supporting the hypothesis that TRI MS are expanding the percentage of Tregs to overall CD3^+^ T cells. To further test whether TRI MS were preventing signs of DED through the expansion of Tregs, anti-GITR was administered to impair Treg function^38^. Specifically, treatment with the agonistic anti-GITR monoclonal antibody (DTA-1) both inhibits the suppressive function of Tregs (via signaling through GITR on Tregs)^39,58,59^ and causes effector CD4^+^ and CD8^+^ T cells (effector T cells) to become resistant to Treg-mediated suppression (via signaling through GITR on conventional T cells)^39,59,60^. We observed that anti-GITR reversed the beneficial effects of the TRI MS (Figs 2A–C and 3B). Also, there was an observed increase in the overall numbers of CD3^+^ T cells, and a substantial reduction in the percentage of FoxP3^+^ of overall CD3^+^ T cells in the lacrimal gland, potentially indicating that Tregs may be needed to mediate the therapeutic effects of TRI MS. Ultimately, the current study provides proof-in-principle for prevention of DED through induction of endogenous Treg in an experimental murine model. Future studies would be geared toward evaluating this drug-delivery strategy as a treatment for pre-existing DED, as well as testing the efficacy of the TRI MS in a pre-clinical larger animal model such as rabbits. Additionally, future studies geared toward translation would determine pharmacokinetic distribution of factors (which would need to be radiolabeled in order to distinguish them from endogenously produced factors) dose-dependent toxicity, and the exploration of other modes of delivery such as subconjunctival and topical placement. One possible mode of delivery that would be relevant for treating patients may be to use new thermogelling formulations that can provide long-term topical delivery to the surface of the eye^61^.

In conclusion, our data suggest TRI MS were able to reduce the local pro-inflammatory milieu of the lacrimal gland tissue. TRI MS prevented tear loss, preserved goblet cell density and reduced corneal fluorescein staining, which indicate that the therapy prevented signs of DED. Importantly, TRI MS were able to decrease the total number of CD3^+^ T cells infiltrating the lacrimal gland tissue and enhance the frequency of FoxP3^+^ T cells among infiltrating T cells. Ultimately, this experimental murine study provides one potential strategy for future anti-inflammatory therapies to focus on harnessing Tregs to restore the local immunological homeostasis within the ocular tissue.

## Electronic supplementary material

Supplemental Dataset
