# Supplementary material for: TRI Microspheres prevent key signs of dry eye disease in a murine, inflammatory model
Source: Sci Rep. 2017 Dec 13;7:17527. doi: 10.1038/s41598-017-17869-y (PMC5727478; doi:10.1038/s41598-017-17869-y)
Supplement: Supplementary file 1 — Supplemental Dataset [file 41598_2017_17869_MOESM1_ESM.doc]

**TRI Microspheres prevent key signs of dry eye disease in a murine, inflammatory model**

**Michelle L. Ratay^a^, Stephen C. Balmert^a^, Abhinav P. Acharya^b^, Ashlee C. Greene^b^, Thiagarajan Meyyappan^b^, and Steven R. Little^a,b,c,d,e,1^**

^a^Department of Bioengineering, University of Pittsburgh, Pittsburgh, PA 15261

^b^Department of Chemical Engineering, University of Pittsburgh, Pittsburgh, PA 15216

^c^Department of Immunology, University of Pittsburgh, Pittsburgh, PA 15213

^d^Department of Ophthalmology, University of Pittsburgh, Pittsburgh, PA 15213

^e^Department of Pharmaceutical Science, University of Pittsburgh, Pittsburgh PA 15261

^1^To whom correspondence may be addressed. Email: [srlittle@pitt.edu](mailto:srlittle@pitt.edu)


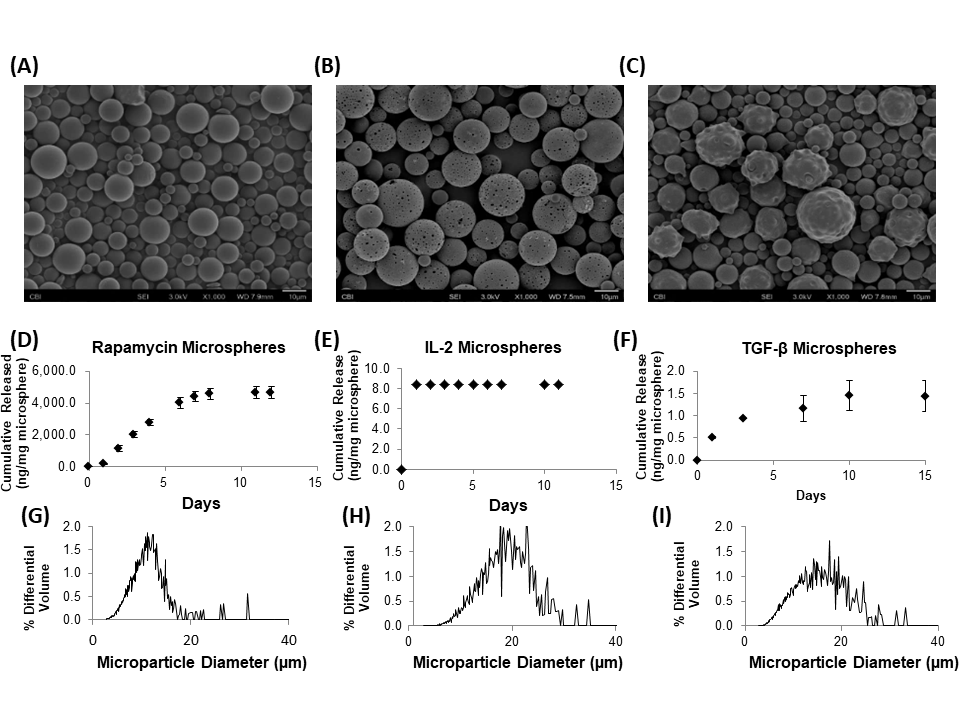


Figure S1. Characterization of Treg-inducing Microspheres (A) Representative Scanning electron microscopy (SEM) image of Rapamycin microspheres (1000x)

(B) Representative SEM image of IL-2 Microspheres (1000x) (C) Representative image of TGF-β Microspheres. (D) Release Kinetics of Rapamycin Microspheres is shown (n=3) (E) Release Kinetics of porous IL-2 Microspheres (n=3) (F) Release Kinetics of TGF-β Microspheres (n=3). (G) Size distribution of Rapamycin Microspheres

(H) Size distribution of IL-2 Microspheres (I) Size Distribution of TGF-β Microspheres


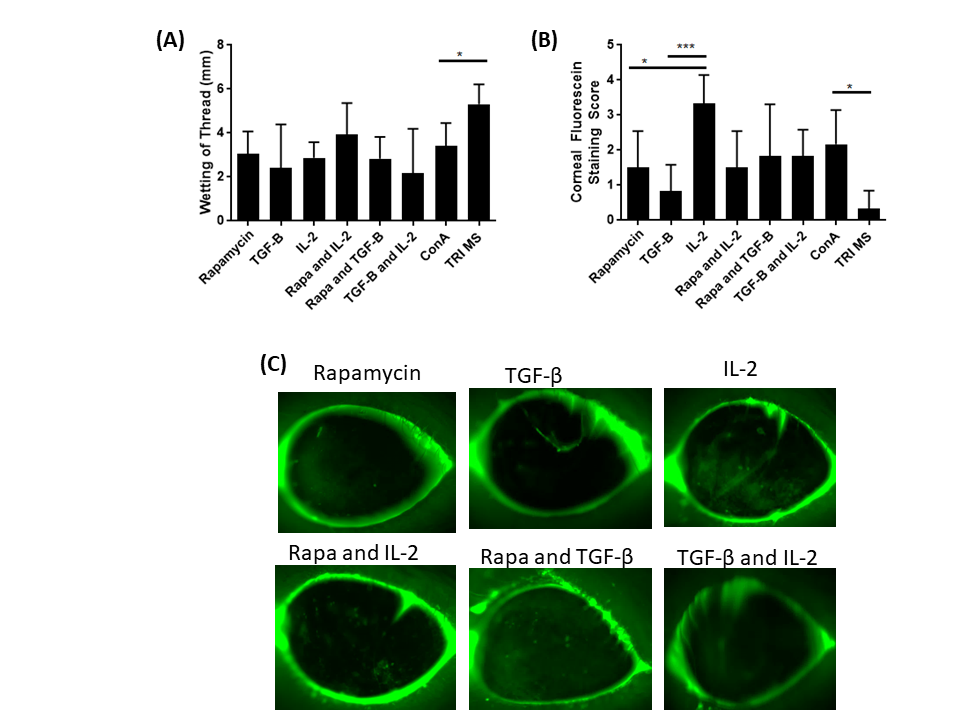


Figure S2. Corneal Fluorescein Staining was evaluated for single factors (Rapamycin; IL-2; TGF-β) and the combination of two factors (Rapa + TGF-β; Rapa + IL-2; TGF-β + IL-2). (A) Phenol Red Thread testing for the single factor (Rapamycin; IL-2; TGF-β) and combination of two factors (Rapa + TGF-β; Rapa + IL-2; TGF-β + IL-2) experimental groups (n=6) as mean ± S.D. * p ≤ 0.05 (B) Ocular Surface Staining score shown as mean ± S.D.* p ≤ 0.05;** p ≤ 0.01; *** p ≤ 0.001. (n=6)

(C) Representative images of corneal fluorescein staining.


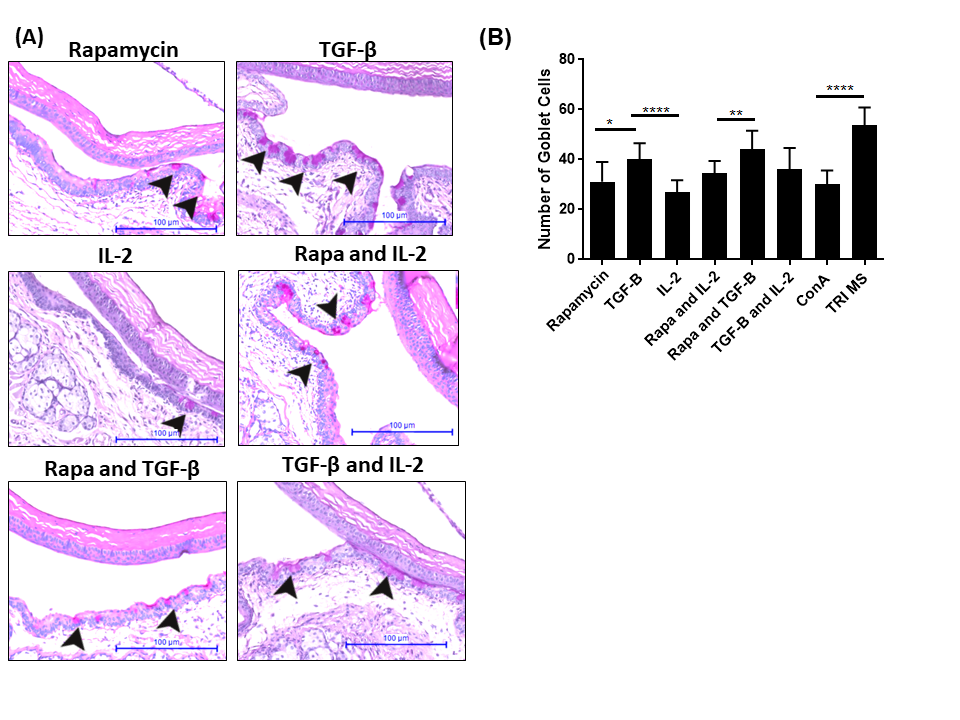


Figure S3. Single factors (Rapamycin; IL-2; TGF-β) and combinations of two factors (Rapa + TGF-β; Rapa + IL-2; TGF-β + IL-2) were utilized to examine goblet cell density in the conjunctiva. (A) Representative images of single and combination factors of PAS stained goblet cells in the conjunctiva (B) Quantification of goblet cell numbers from the histology of the conjunctiva are shown as mean ± S.D. * p ≤ 0.05;** p ≤ 0.01; *** p ≤ 0.001, **** p ≤ 0.0001.
